# Decreased Laminin Expression by Human Lung Epithelial Cells and Fibroblasts Cultured in Acellular Lung Scaffolds from Aged Mice

**DOI:** 10.1371/journal.pone.0150966

**Published:** 2016-03-08

**Authors:** Lindsay M. Godin, Brian J. Sandri, Darcy E. Wagner, Carolyn M. Meyer, Andrew P. Price, Ifeolu Akinnola, Daniel J. Weiss, Angela Panoskaltsis-Mortari

**Affiliations:** 1 Department of Pediatrics, University of Minnesota Medical School, Minneapolis, Minnesota, United States of America; 2 Department of Medicine, University of Minnesota Medical School, Minneapolis, Minnesota, United States of America; 3 Department of Medicine, University of Vermont, Burlington, Vermont, United States of America; 4 MSTP Program, University of Minnesota Medical School, Minneapolis, Minnesota, United States of America; National Institute of Environmental Health Sciences, UNITED STATES

## Abstract

The lung changes functionally and structurally with aging. However, age-related effects on the extracellular matrix (ECM) and corresponding effects on lung cell behavior are not well understood. We hypothesized that ECM from aged animals would induce aging-related phenotypic changes in healthy inoculated cells. Decellularized whole organ scaffolds provide a powerful model for examining how ECM cues affect cell phenotype. The effects of age on ECM composition in both native and decellularized mouse lungs were assessed as was the effect of young vs old acellular ECM on human bronchial epithelial cells (hBECs) and lung fibroblasts (hLFs). Native aged (1 year) lungs demonstrated decreased expression of laminins α3 and α4, elastin and fibronectin, and elevated collagen, compared to young (3 week) lungs. Proteomic analyses of decellularized ECM demonstrated similar findings, and decellularized aged lung ECM contained less diversity in structural proteins compared to young ECM. When seeded in old ECM, hBECs and hLFs demonstrated lower gene expression of laminins α3 and α4, respectively, as compared to young ECM, paralleling the laminin deficiency of aged ECM. ECM changes appear to be important factors in potentiating aging-related phenotypes and may provide clues to mechanisms that allow for aging-related lung diseases.

## Introduction

Aging is known to be associated with structural changes in extracellular matrix (ECM), and ECM dysregulation was recently proposed to be a hallmark of aging in the lung [1]. In the lung, the functional consequence is manifested primarily as decreased elasticity [2]. Although much interest has focused on the roles of oxidative stress, stem cell senescence, autophagy, defective mitochondrial function, and inflammasome production by aging cells, the effects of age-related changes in lung ECM on normal cell behavior remains less well understood. Aging-related ECM changes may also contribute to aging-associated lung diseases such as idiopathic pulmonary fibrosis (IPF), chronic obstructive pulmonary disease (COPD) and senile emphysema [3]. In mouse and human lungs, decreased elastin content precedes the increase in collagen with age [4, 5]. Increased degradation and release of elastin peptides leads to uncoupling of the elastin-laminin receptor and alters signal transduction in parenchymal cells [6]. In murine models, the aged lung is more sensitive to fibrotic injury [7]. However, there are limited studies which directly examine how the underlying changes in ECM contribute to changes in cellular phenotype. This has largely been in part due to limited *in vitro* and *in vivo* tools with which to address these questions[8].

Lung decellularization provides a novel tool to assess the specific changes in ECM composition in aged lungs and to assess the effect of aging on phenotype and behavior of inoculated cells. A previous study used mass spectrometry to assess protein content of decellularized old vs young mouse lungs [9]. Several ECM proteins, particularly laminins, were found to be significantly decreased in old lungs, yet there was no obvious difference in recellularization of normal young versus aged tissue with mouse stromal or alveolar epithelial cells (albeit an immortalized cell line). Cell survival was impaired in decellularized lungs from aged mice with elastase-induced emphysema suggesting that disease processes in aged lungs may magnify any age-related changes. However, the effect of the matrix on the cell phenotypes was not assessed and the mass spectrometry proteomics approach utilized was limited to readily-soluble and the most abundant proteins.

The goal of this study was to further determine age-related changes in the ECM proteins in mouse lungs. In addition to biochemical assays, two different mass spectrometry proteomic methods, including quantitative iTRAQ, were used to better assess residual ECM proteins remaining in decellularized old vs young mouse lungs. Finally, the effects of aged vs young ECM on the phenotype of human lung epithelial cells and fibroblasts inoculated into decellularized mouse lungs, ventilated in bioreactors, were assessed.

## Methods

### Animals

Male and female B10.BR (4do, 3wo, 3mo and 1yo) and BALB/c (3wo and 2yo) mice were bred (original breeders from Jackson Labs, Bar Harbor, ME) and housed in microisolator cages in specific-pathogen-free housing at the University of Minnesota. Mice were euthanized with Nembutal. The use of mice was approved by the University of Minnesota’s Institutional Animal Care and Use Committee.

### Lung Tissue Decellularization

Details of the decellularization protocol have been previously published [10]. The lungs were sequentially perfused via the airways and the vasculature with distilled water, Triton X-100, sodium deoxycholate, NaCl, DNase and PBS.

### Cells

Normal hBECs, from a male donor in their 60’s, were purchased from Lonza (Portsmouth, NH, USA) were grown in basal bronchial epithelial cell growth medium (BEGM) with the BEGM bullet kit (Lonza). Primary hLFs were isolated as described [11], (from excess de-identified control patient lung tissue of a female in their 70’s, whose use was approved by the University of Minnesota Institutional Review Board IRB#0611E97107), grown in DMEM, 10% FBS, and 1% P/S, and used at Passage 2. The cells were grown to confluence, trypsinized, and counted for inoculation into decellularized lungs. Human cells were utilized so that inoculated cell contributions to matrix remodeling could be distinguished from the pre-existing mouse matrix composition.

### Bioreactor Setup and Culture

Prior to cell injection, acellular lungs were rinsed through the vasculature and the trachea with cell-specific medium. Decellularized lungs from 3 week and 1 year old mice were cannulated via the trachea with an appropriately sized cannula (modified 22- or 19-gauge needle, respectively) and placed into a T25 flask containing media as described [10]. HBEC or hLF cells were manually injected via the cannula with a 1mL syringe and 19x1 ½ gauge needle at a concentration of 7.5 million cells in 0.3 mL of medium for the 3 wo, and 15 million cells in 0.5 mL of medium for the 1yo scaffolds (to account for size difference as 1 yo lungs are approximately twice the size of 3 wo lungs). No leakage of cells was observed from any scaffold. After 1 hour of static culture to allow cell adherence, lungs were ventilated (Kent Scientific, Torrington, CT) in normal mode for 1 week at a physiological rate of 75 breaths per minute, PEEP at 3 cm H_2_O, and 28cm H_2_O peak pressure, with inspiration set at 45% and a tidal volume of approximately 100 and 225 uL for 3wo and 1yo lungs, respectively. Fresh media (50 uL &100 uL for 3wo and 1yo lungs, respectively) was infused down the trachea every other day (ventilation was briefly interrupted). The experiment was repeated 3 times for each cell type and each age.

### Extracellular Matrix Assays

ECM assays were done on 4do, 3wo, 3mo and 1yo native and decellularized lungs (n = 6/group). For the hydroxyproline assay, the superior (upper) lobe of the right lung was weighed after drying overnight at 60°C to determine the dry weight, and OH-proline content determined on hydrolyzed samples (6N HCl) at 560nm [10].

Elastin content was measured with a Fastin Elastin Assay kit (Biocolor Life Sciences, Carrickfergus, United Kingdom) on post-caval right lobe samples hydrolyzed in 0.25M oxalic acid. Total laminin, and vitronectin contents were determined from right middle lobe (**RML**) samples homogenized in 0.9% NaCl and 0.2% Triton X-100, using kits from Insight Genomics (Falls Church, VA), and Molecular Innovations (Novi, MI), respectively. From the same RML, sulfated glycosaminoglycan GAG content was determined through generation of the GAG-DMMB (dimethylmethylene blue) complex and measured at 656nm with a 540nm correction factor [12]. Total protein content of the RML was determined by the Bradford method.

### Histology and Immunofluorescence

Haematoxylin and eosin (H&E), and trichrome (collagen) stains were used to histologically examine cryosections of native and decellularized left lungs (inflated as described[10]). A Verhoeff-Van Gieson Elastic Stain Kit (Sigma-Aldrich, St. Louis, MO) allowed for visualization of the distribution of elastin. Sulfated GAGs were detected using Alcian Blue and counterstained with Safranin 0. For other analytes, the antibodies used for immunofluorescence of cryosections are listed in **S1 Table**. Images were taken using an RT-Spot camera on an Olympus BX51 microscope.

### qRT-PCR (native lungs and decellularized/recellularized lungs)

Total RNA from inferior (lower) right lobes was prepared using Trizol (Life Technologies, Grand Island, NY) and the PureLink™ RNA MiniKit (Life Technologies), and cDNA generated with the Superscript III kit (Invitrogen). Quantitative real-time PCR was performed on an ABI 7500 Real Time PCR System using TaqMan® Gene Expression Master Mix (Applied Biosystems). Probes were purchased from Life Technologies (Grand Island, NY) and are listed in **S2 Table**. For native lung evaluations, n = 6/age group. For recellularization of acellular lungs, the experiment was repeated 3 times for each cell type and each age (3wo and 1yo). Each sample was run in duplicate. Samples were considered negative for a particular mRNA if >35 cycles were required for detection. Successful initial decellularization was confirmed by negative genomic PCR for murine GAPDH.

### Mass Spectrometry

Since mass spectrometry data is largely dependent on the digestion and separation techniques, two different methods were compared. Decellularized left lungs were analyzed using a conventional semi-quantitative (non-labeled) method as well as a quantitative iTRAQ (labeled) method [13]. Details are provided in **S1 Supplemental Methods**.

### Statistical Analyses (quantifiable, non-Mass Spec data)

Statistical comparisons were done between ages within native lung groups, or between decellularized lung age groups, not between native and decellularized groups. An F-Test of Variance was performed to determine if the variances were significantly different between groups. If the test was not significant, a standard T-Test was used. If variances were significantly different, a T-Test assuming unequal variances was run. All numerical data are displayed as means ± standard errors.

## Results

### Age-related changes in matrix composition

**Fig 1:**
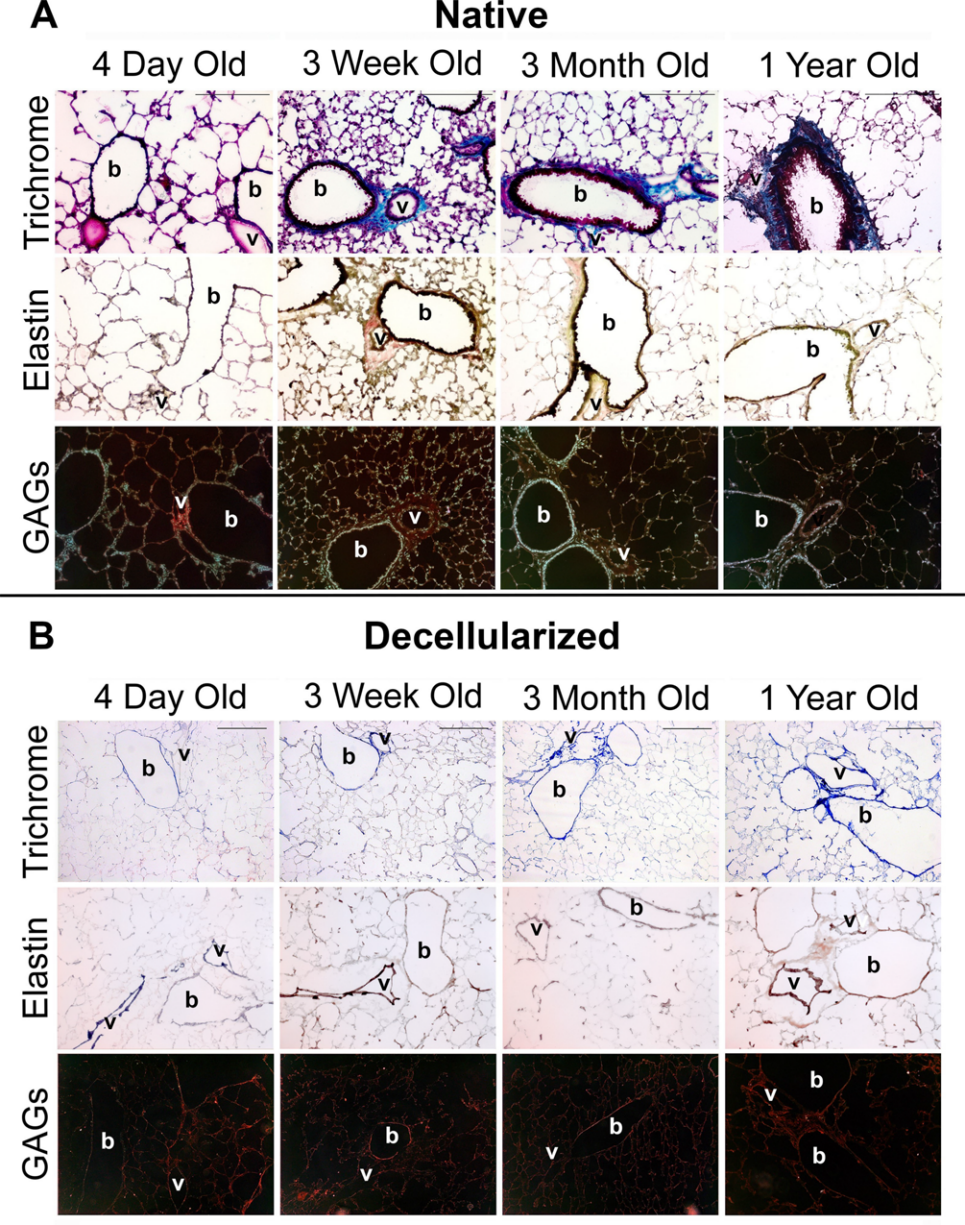
**Matrix stains of native (A) and decellularized (B) lungs of mice of different ages.** Stains for Trichrome (collagen is blue), elastin (black) and GAGs (red) are shown. Representative images are shown for 1 out of 3 mice per age group. V = blood vessel; b = bronchiole. Magnification 200X, size bars = 200um (size bars on top row apply to all rows).

We initially qualitatively determined the ECM composition in native and decellularized lungs from B10.BR mice over a wide age range from 4 days to 1 year of age (**Fig 1**). In native lungs (**Fig 1A**), the amount of overall total collagen (blue) appears least abundant in the youngest age group while total elastin (black) appears least abundant in the oldest age group. As in previous studies, normal histologic appearance of the branching architecture was maintained after decellularization (**Fig 1B**). The intensity of staining for collagen (Trichrome-blue) increased with age in the decellularized lungs in all areas. Elastin and GAGs, although present, were the most affected by decellularization as seen by decreased staining intensity compared to native lungs. The decrease in GAGs may in large part reflect the loss of cell-associated GAGs after decellularization, but no apparent differences were seen across age groups. The majority of the elastin staining in decellularized lungs was seen in the vascular (v) and bronchiolar (b) walls.

**Fig 2:**
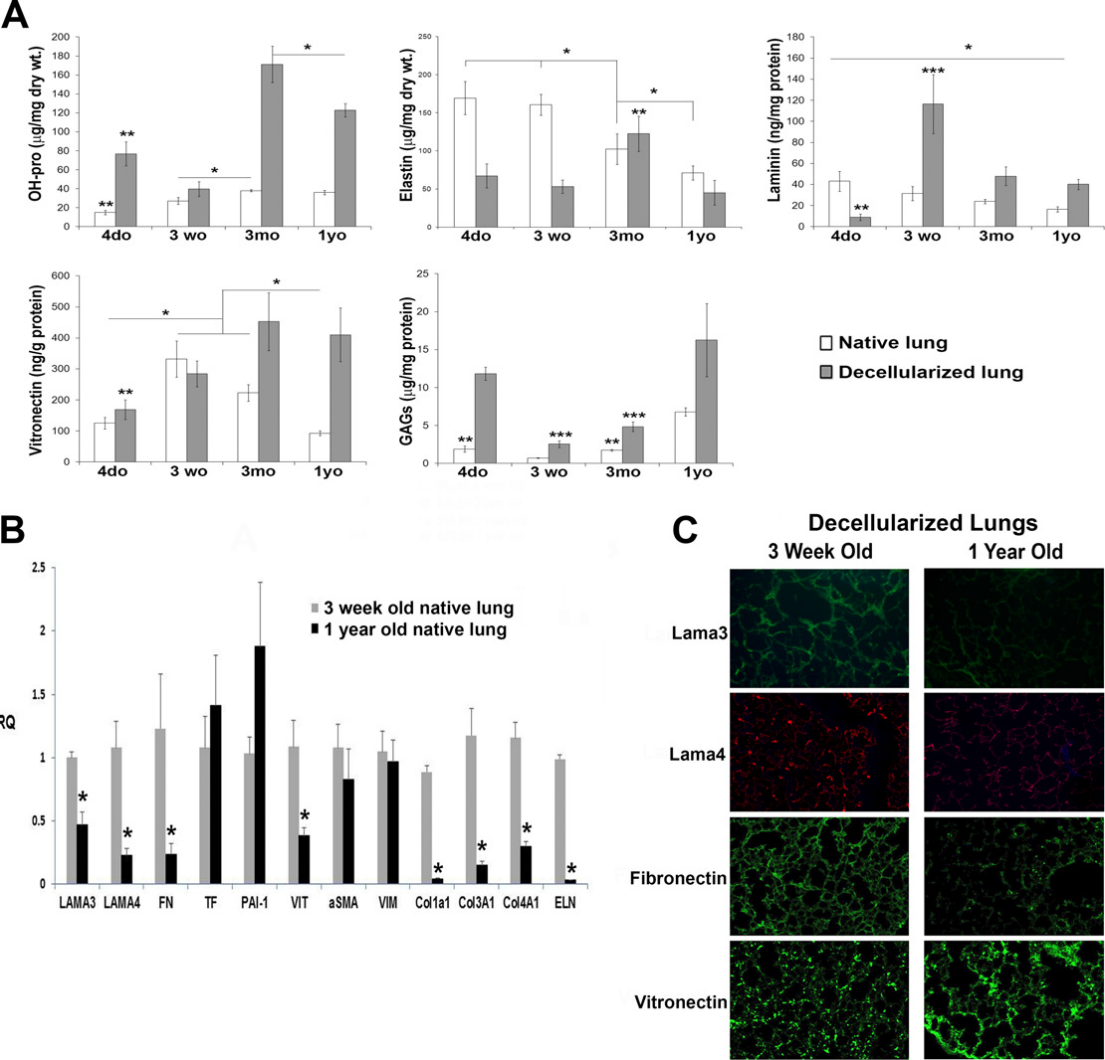
Differential expression of matrix-associated genes and proteins in young versus old mouse lungs. **A**) Comparison of ECM component levels in native and decellularized lungs of different aged B10.BR mice. Statistical comparisons were done only between ages within a treatment group, not between treatment groups. *p<0.05 comparing ages indicated by bar. N = 6 for all groups. For hydroxyproline, **p<0.05 for 4 do vs. all other ages in treatment group; for elastin, **p<0.05 for 3 mo decell vs. other decell ages; for total laminin, **p<0.05 for 4 do decell vs. other decell ages, ***p<0.05 for 3 wo decell vs. other decell ages; for vitronectin, **p<0.05 for 4 do decell vs. other decell ages; for sulfated Glycosaminoglycans, **p<0.05 for 4 do or 3 mo non-decell vs. 3 wo or 1 yo non-decell, ***p<0.05 for 3 wo or 3 mo decell vs. 4 do and 1 yo decell. Abbreviations used: do = day old, wo = week old, mo = month old, yo = year old. **B)** Gene expression in native lungs from young and old B10.BR mice as assessed by qRT-PCR. N = 6/group; *p<0.05 old vs young. Values are normalized to a representative 3 wo mouse. **C)** Immunofluorescence images of mouse Lama3, Lama4, fibronectin and vitronectin staining of decellularized lungs from 3-week vs 1-year old B10.BR mice. Magnification 200X. Images are 1 representative of 3 decellularized lungs per group.

Quantitative measures of hydroxyproline (collagen), elastin, total laminin, total vitronectin, and sulfated GAGs were determined for all ages for both native lung and decellularized lung matrix (from the same sets of lungs used for Fig 1) (**Fig 2A)**. Collagen significantly increased with age in both the native and decellularized lungs. As expected, decellularization resulted in an increase in the proportion of collagen per unit dry weight of lung. Elastin significantly decreased with age in the native lung but its proportion to unit dry weight did not increase by decellularization indicating that decellularization removes some elastin. Among the decellularized samples, elastin level was the highest in the 3mo lungs. Total laminin decreased significantly with age in the native lung. In decellularized lungs, 3wo lungs had the highest laminin levels with decreases in older ages. Vitronectin levels peaked at 3 weeks of age in the native lungs and were enriched in decellularized 3mo and 1yo lungs. Sulfated GAG levels in native lungs were highest in 1 yo lungs and were enriched in all age groups by decellularization (proportionally to the age-matched native lungs) with the highest levels in 4do and 1yo lungs.

The age-related decrease in laminin was corroborated by quantitative RT-PCR of native lungs. **Fig 2B** shows that expression of both laminin α3 (LAMA3) and α4 (LAMA4) mRNA was significantly decreased in aged native B10.BR lungs. Similar decreases were found in aged BALB/c mice, showing that age-related changes were not strain-specific (**S1 Fig**). Decreases in laminin were accompanied by decreased mRNA expression for elastin (ELN), fibronectin (FN), vitronectin (VIT), and collagens 1, 3 and 4 (**Fig 2B**). In contrast, mRNA levels of PAI-1, a cellular protein associated with aging and also with fibrosis, was elevated, albeit not to a statistically different degree in the 1yo B10.BR mice, but was significantly higher in the 2yo BALB/c mice (**S1 Fig**). Since 3wo and 1yo lungs were identified to be disparate with respect to their ECMs as determined above (and due to difficulty in handling 4do lungs), only these 2 age groups were studied in all subsequent analyses. Furthermore, although 1yo mice are considered midlife and not aged, it has been shown that there is a critical time period between the age of 8 months and 12 months where there is a transition in lung structure as evidenced by airspace enlargement, oxidative stress and cell death, as well as immune cell activation heralded by immune complex deposition[14]. This lends credence to our study of ECM at this pre-senescent stage of 1 year of age and the comparison to the younger 3 week age.

Due to differences in the transcript levels of Laminins α3 & α4, fibronectin, and vitronectin in 3wo and 1 yo B10.BR mouse lungs, protein level differences were examined qualitatively in decellularized lungs using immunofluorescence staining to see how well these differences translated to the decellularized extracellular matrix. Qualitative decreases were observed in Laminins α3 & α4, as well as fibronectin in the 1 yo decellularized lungs (**Fig 2C**) compared to 3wo lungs. Despite the decrease in mRNA for vitronectin, the staining intensity for vitronectin in 1yo decellularized lungs was higher than in 3wo lungs (**Fig 2C**, bottom panels), consistent with the findings of **Fig 2A** for decellularized lungs. The staining pattern for vitronectin in the 1yo decellularized lungs also appeared less evenly distributed than in the 3wo lungs.

Owing to methodological differences in protein identification using mass spectrometry proteomics, both a standard 1D in-gel trypsin digestion method[9], and iTRAQ[13] method were used for proteomic analysis of decellularized young and old lung ECM. Significant age-related differences were found in several ECM proteins as well as other proteins remaining in the scaffold, mostly cytoplasmic, cytoskeletal, and mitochondrial in origin. Both methods resulted in identifying many differentially expressed proteins. In comparing the 2 most disparate age groups by the methods described above (i.e. 3wo vs 1yo), the young, 3wo lungs expressed greater diversity in the type and variety of structural proteins detected compared to the older, 1yo lungs (**S3 Table**). To better characterize the changes in ECM composition, we used the well-characterized iTRAQ reagent which provides true quantitative assessment of a wider range of proteins [13]. Significant proportional increases of several ECM proteins, mostly collagens, as well as nephronectin, were observed in old lungs, compared to young lungs (**Table 1**). Aged lungs demonstrated decreases in many laminin chains (α2, α3, α4, α5, β1, β2, β3, γ1, γ2) as well as several other ECM proteins listed in **Table 1**.

**Table 1:** Differentially expressed ECM proteins in young versus old decellularized murine lungs identified by iTRAQ.

Gene Symbol	Gene Name	Old/Young
Agrn	Agrin	0.34
Col1a1	Collagen alpha-1(I) chain	1.71
Col1a2	Collagen alpha-2(I) chain	1.75
Col2a1	Collagen alpha-1(II) chain	4.00
Col3a1	Collagen alpha-1(III) chain	1.29
Col4a1	Collagen alpha-1(IV) chain	4.61
Col4a2	Collagen alpha-2(IV) chain	2.67
Col4a3	Collagen alpha-3(IV) chain	3.94
Col5a1	Collagen alpha-1(V) chain	2.06
Col5a2	Collagen alpha-2(V) chain	2.17
Col5a3	Collagen type V alpha 3 chain	2.60
Col6a1	Collagen alpha-1(VI) chain	0.70
Col6a2	Collagen alpha-2(VI) chain	1.07
Col6a5	Collagen alpha-5(VI) chain	2.29
Col6a6	Collagen alpha-6(VI) chain	1.86
Col7a1	Collagen alpha-1(VII) chain	0.38
Col12a1	Collagen alpha-1(XII) chain	0.42
Col18a1	Isoform 3 of Collagen alpha-1(XVIII) chain	2.77
Dcn	Decorin	0.26
Eln	Elastin	0.48
Emilin1	EMILIN-1	0.41
Fgb	Fibrinogen beta chain	0.81
Fn1	Fibronectin	0.58
Fbln5	Fibulin-5	0.81
Hspg2	Basement membrane heparan sulfate proteoglycan core	0.57
Lama3	Laminin subunit alpha-3	0.50
Lama5	Laminin subunit alpha-5	0.70
Lamb2	Laminin subunit beta-2	0.98
Lamc1	Laminin subunit gamma-1	0.73
Lamb3	Laminin subunit beta-3	0.41
Lamc2	Laminin subunit gamma-2	0.31
Lama2	Laminin subunit alpha-2	0.50
Lamb1	Laminin subunit beta-1	0.28
Lama4	Laminin subunit alpha-4	0.88
Mfap4	Microfibril-associated glycoprotein 4	0.38
Nid2	Nidogen-2	0.58
Npnt	Nephronectin	1.14
Postn	Periostin	0.34

### Aged matrix regulates phenotype and ECM production in human lung epithelial and fibroblast cells

**Fig 3:**
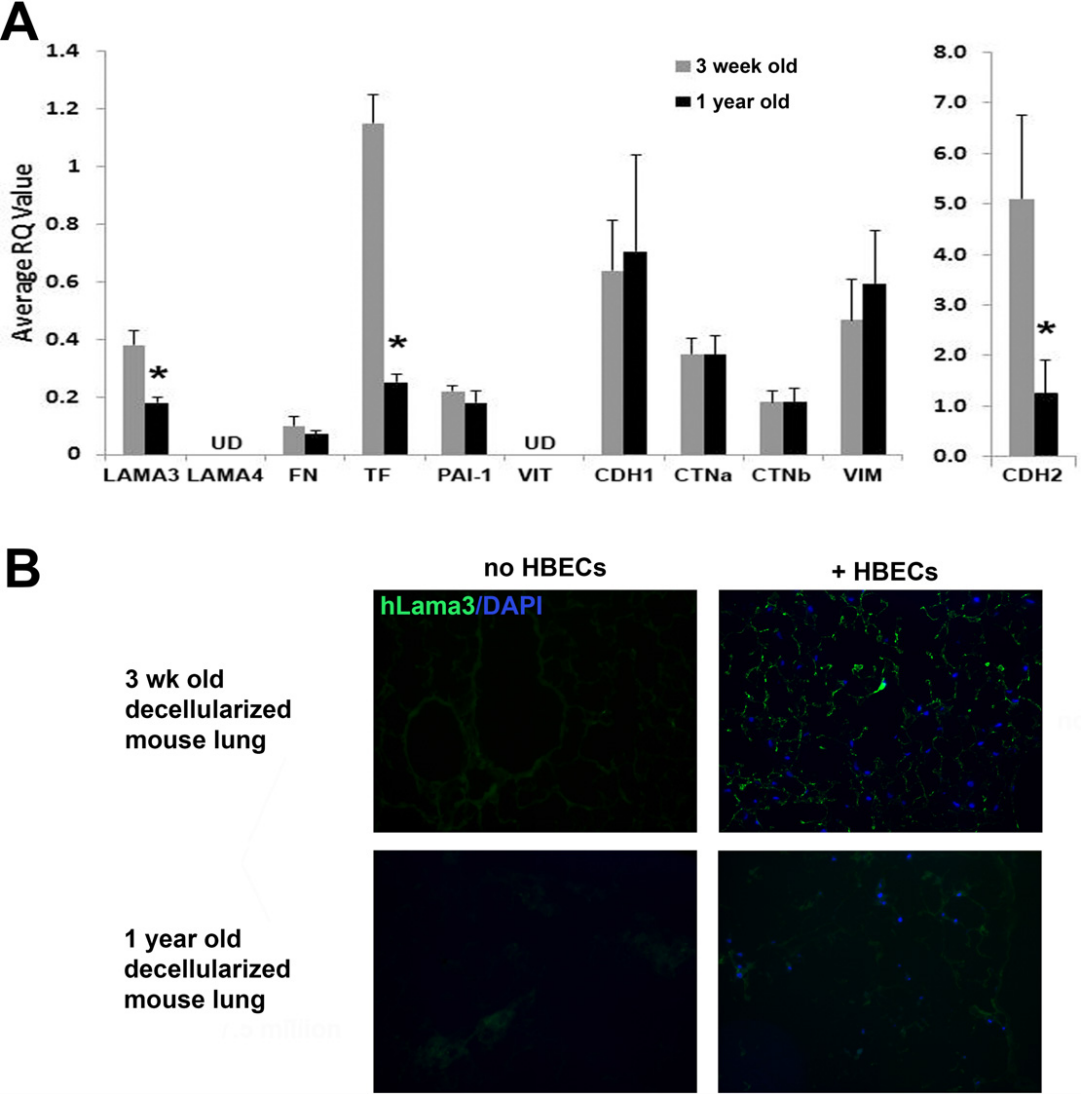
Decreased expression of laminin α3, tissue factor and N-cadherin in human bronchial epithelial cells incubated in old versus young lung matrix. **A)** Gene expression (qRT-PCR) of hBECs after 1 week incubation in young and old decellularized B10.BR mouse lung matrix. N = 3/group; *p<0.05 for 1 yo vs 3 wo. Data are normalized against hBECs grown on tissue culture plastic. UD = undetected. **B)** Immunofluorescence staining shows decreased human Lama3 deposition (green) by hBECs infused in 1yo decellularized lung matrix compared to 3wo matrix. Non-infused, age-matched, decellularized lungs are shown as controls. DAPI nuclear staining is shown in blue. Distal alveolar regions are shown. Magnification 200X. Images are 1 representative of 3 lung matrices per condition.

To evaluate the effect of lung matrix age on the behavior of human lung cells, decellularized lungs from young (3 wo) and old (1yo) mice were inoculated with hBECs or hLFs through the airway and incubated for 1 week in bioreactors with mechanical ventilation. When grown on aged lung matrix, hBECs had decreased expression of laminin-α3, tissue factor, and N-cadherin (CDH2) mRNA compared to cells grown on young matrix, with no significant changes in fibronectin, PAI-1, E-cadherin (CDH1), α & β catenin (CTNa/CTNb), or vimentin (**Fig 3A**). No collagen mRNA was detected as expected. HBECs were also negative for laminin-α4, as expected, since fibroblasts, not epithelial cells, are the major producers of laminin-α4. Tissue constructs composed of decellularized matrix and inoculated hBECs were also analyzed qualitatively for laminin-α3 by immunofluorescence after 1 week incubation in ventilated bioreactors. **Fig 3B** shows that hBECs produce less human laminin-α3 when incubated in 1yo ECM compared to 3wo ECM.

**Fig 4:**
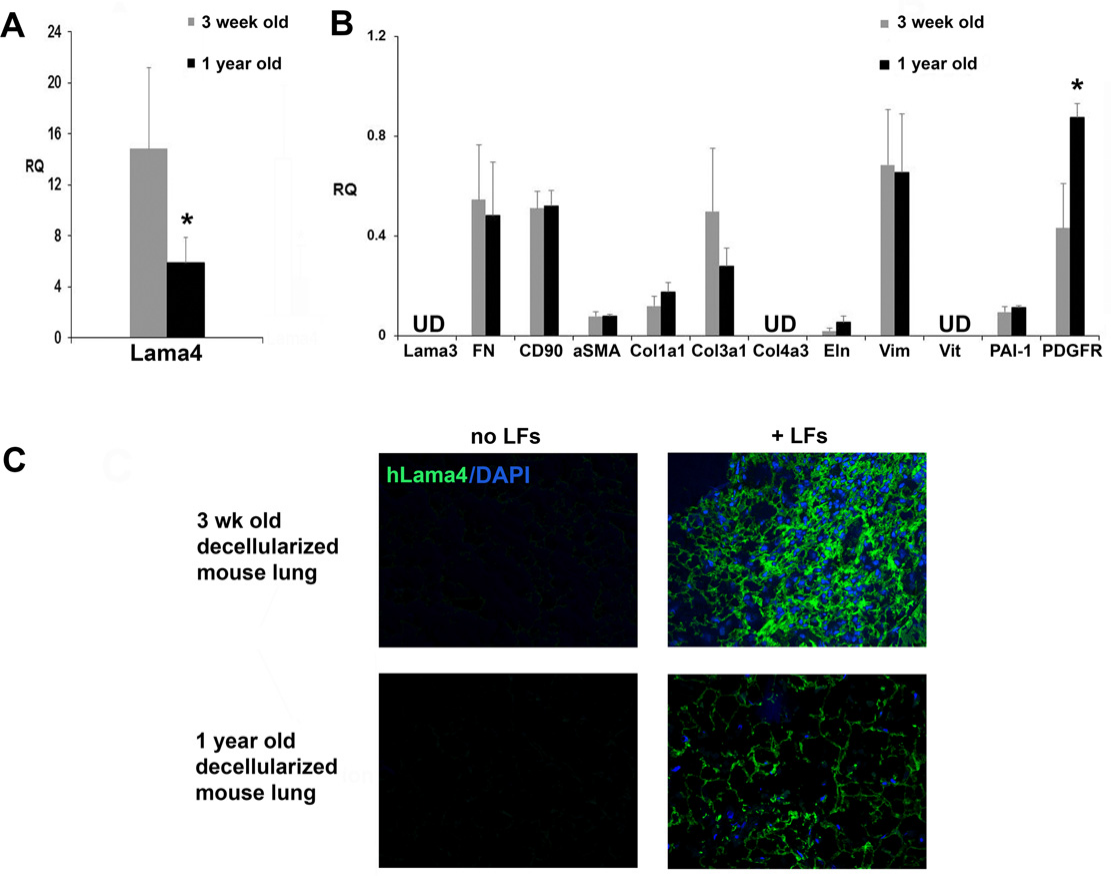
Human LFs deposit less laminin α4 in old lung matrix compared to young lung matrix. HLFs were incubated for 1 week in decellularized B10.BR mouse lungs. **A)** and **B)** Gene expression (qRT-PCR) of hLFs after 1 week incubation in young and old decellularized BALB/c mouse lung matrix. N = 3/group; *p<0.05 for 2 yo vs 4 wo. All values in **A)** and **B)** were normalized to cells grown on tissue culture plastic. UD = undetected. **C)** Immunofluorescence staining shows decreased human laminin α4 deposition (green) by hLFs infused in 1yo decellularized lung matrix compared to 3wo matrix. Non-infused, age-matched, decellularized lungs are shown as controls. DAPI nuclear staining is shown in blue. Distal alveolar regions are shown. Magnification 200X. Images are one representative of three lung matrices per condition.

When cultured in 1yo aged matrix, hLFs exhibited decreased expression of mRNA for laminin-α4, compared to hLFs grown on young 3wo matrix (**Fig 4A**). No significant changes in collagen or fibronectin mRNA were found when hLFs were cultured in 1yo ECM (**Fig 4B**) but decreases were apparent for these mRNAs as well as for CD90 (Thy-1 marker) and αSMA when hLFs were cultured in 2yo ECM (**S2 Fig**, albeit in a different mouse strain). In contrast, PDGFR expression increased in hLFs grown on 1yo aged matrices (**Fig 4B**). HLFs were negative for laminin-α3, as expected, since epithelial cells, not fibroblasts, are the major producers of laminin-α3. Tissue constructs composed of decellularized matrix and inoculated hLFs were also qualitatively analyzed by immunofluorescence after 1 week incubation in ventilated bioreactors. **Fig 4C** shows that hLFs qualitatively produce less human laminin-α4 when incubated in old ECM compared to young ECM.

## Discussion

This study demonstrated that decreased laminin production is an early hallmark of the aging lung. Through proteomic analyses, we found that young lung ECM expressed greater diversity in the type and variety of structural proteins detected compared to old lung ECM. Notably, decellularizing lungs from different ages of mice resulted in acellular scaffolds with differing amounts and ratios of ECM components. Compared to young ECM, old acellular ECM caused lower levels of laminin transcription in human lung epithelial cells and fibroblasts. This is a novel finding that provides further insight into the effect of aged ECM on cell behavior, and also suggests that this is a good model system for further probing the matrix-based mechanisms affecting laminin production by these cells.

Laminins are trimeric ECM molecules (α, β and γ subunits), that are important in tissue development. Although much is known about their roles in lung development [15], little is known about their roles in aging. Laminin-α3 (part of laminin-5 and-6 isoforms) is predominantly made by epithelial cells [16]. One of the most striking findings observed with ECM gene expression in native lungs was the age-related decrease in laminin α3, and decreased expression of laminin α3 by HBECs exposed to acellular old, compared to young, lung ECM,. Loss of laminin-α3 in murine lungs is associated with increased collagen and resistance to mechanical injury [17]. The associated decreased fibronectin that was observed in aged native lungs may be relevant to the role of fibronectin in supporting migration of bronchial epithelial cells on laminin-α3 [18], hence affecting the capacity for epithelial repair.

HLFs demonstrated significantly decreased expression of human laminin-α4 when they were incubated on aged, versus young, mouse lung ECM. It has been previously shown that the ECM of decellularized lungs from aged mice and human IPF express decreased laminin-α3 and -α4 [9, 19]. Laminin-α4 is primarily produced by mesenchymal and endothelial cells [16]. Its deficiency destabilizes the vasculature [20]. Furthermore, Laminin-α4 down-regulates the PDGF receptor [21] through which PDGF normally signals an increase in collagen production. Knockout of laminin-α4 in mice results in increased collagen deposition and kidney fibrosis associated with increased PDGF and PDGFR expression[21]. Indeed, we found that when cultured in 1yo lung ECM containing low levels of laminin-α4, hLFs expressed increased PDGFR. Human LFs also exhibited decreased mRNA for CD90 (Thy-1 marker) when grown on ECM of the even more age-advanced 2yo lungs. CD90-negative fibroblasts are considered to be profibrotic [22] and are the predominant fibroblast population in the lungs of aged mice [7] that promote myofibroblast differentiation [23]. Paradoxically, αSMA mRNA was decreased in hLFs grown on old ECM implying that other non-ECM signals are required for myofibroblast differentiation, although ECM from human IPF lungs can induce a profibrotic transcriptome in fibroblasts from non-fibrotic lungs[24].

Others have shown that aged tissue ECM exhibits many changes compared to young ECM. Collagen I is less organized and not as abundant as it is in young ECM [25]. In human lungs with IPF, collagen III content is significantly lower than in normal lungs [26]. Collagen III is part of the finer reticular network that provides tissue compliance [27]. In our study, native lungs from aged mice had decreased expression of mRNA for several collagens, and collagen I expression was completely abrogated in hLFs grown on 2 yo aged ECM, consistent with early studies showing that high collagen levels can attenuate collagen production in human dermal fibroblasts[28]. It may seem paradoxical to have increasing ECM collagen with age in the face of decreased collagen production but this is consistent with the demonstration that collagen has a very long half-life[29], and the collagen fractional degradation rate also decreases with age, resulting in more net ECM deposition[30]. Collagen from aged ECM contains more of the glycosaminoglycan hyaluronan (HA) than does collagen from young ECM [31]. Sulfated GAGs were increased in aged mouse lungs, although the different types of sGAGs weren’t characterized in the native and decellularized lungs in the current study.

Decorin, fibrillin-2, fibulin-5 and emilin-1 were among the several ECM proteins decreased in old lungs. Decorin binds to collagen I fibrils and other components such as fibronectin, and thrombospondin. It is considered as a protective ECM factor because of its ability to downregulate receptor tyrosine kinases and pro-tumorigenic signaling [32, 33]. Fibrillin-2 and fibulin-5 are elastogenesis components needed for elastic fiber formation [34, 35]. The decrease in Emilin-1 in the old ECM is relevant because it too can regulate elastogenesis[36] and can also inhibit TGFβ signaling[37]. This is consistent with the finding of decreased elastin in aged native lungs and also has implications for increased fibrogenesis in aging-related lung diseases.

Beyond age-associated changes in gene expression, it would be interesting to determine whether differential detergent solubility [38, 39] of the tissue is altered with age. Regardless, as stated above, the ability to generate ECM with different compositional profiles provides a good model system to study matrix-based mechanisms of cell behavior as evidenced by the different responses of human lung cells when cultured on young vs old ECM. In studies using other organs, the younger age of ECM scaffolds has been shown to be an important factor for more efficient remodeling and cell repopulation [40, 41]. ECM from young MSCs can rejuvenate old MSCs with respect to their ability to differentiate and can delay senescence[42]. In this study, the human cells were obtained from older donors, and yet, they responded by producing laminin levels relatively consistent with the age of the ECM they were exposed to, revealing the instructive nature of the ECM. This is analogous to what has recently been shown for human lung fibroblast responses on IPF versus control lung ECM[24]. In future studies, it will be interesting to compare the responses of young versus old human lung cells on young and old decellularized lung ECM. There are also reports of age and gender interactions on ECM-associated genes in various tissues in humans[43–46]. The changes in systemic levels of matrix degrading enzymes such as MMPs (some increase, others decrease) with age are more apparent in men than in women[44]. [45]Not much is known about the gender-age interaction with respect to the lung although the effect of gender on lung development (owing to expression of estrogen receptors on lung epithelium) is known[47] and it has been reported that some ECM-associated gene transcription levels exhibit gender-associated trends in IPF and COPD[48]. The serum levels of MMP-9, its inhibitor TIMP-1, and the ratio of the two are inversely correlated with lung function (as assessed by FEV_1_) in men but not women[45]. Matrix degrading enzymes could affect not only cell-matrix interactions but also cell-cell interactions as many growth factors can be released upon degradation and remodeling of the ECM. Reports on the role of gender in the bleomycin mouse model of fibrosis have been conflicting[49–52]. In the current study, no differences were detected in the ECM of decellularized lungs obtained from male and female mice, although the study was not powered to do so. Another limitation of the study is that the response of human cells on aged versus young human (as opposed to mouse) lung ECM was not tested due to the challenges of obtaining non-diseased human lungs of different age groups. The issue of the effect of the mechanical properties of the decellularized lungs was also not directly addressed in this study. Dynamic stretch effects, such as the mechanical ventilation approach used in the current study, may also significantly affect cell behavior through the sensing of scaffold stiffness. Although the transition in lung structure is evident by 1 year of age in mice [14] with altered pulmonary mechanics such as decreased lung tissue elastance and increased airway resistance[53], measurements of local stiffness by atomic force microscopy has not shown differences between young and old mouse lungs after decellularization[54]. Therefore, at the cellular level, the differences in ECM composition of young versus old decellularized mouse lungs may have played the larger role in affecting the human cell responses seen in the current study, although mechanical effects are likely to contribute and cannot be ruled out.

An important parallel outcome of our study is for the implications it has for lung bioengineering using decellularized lung ECM scaffolds. Since ECM composition affects pneumocyte differentiation [55] and aging is a factor in the development of certain lung diseases, the age of the donor of the decellularized organ tissue should be considered in lung bioengineering. However, what is likely more important is to identify the ECM composition most conducive to successful repopulation or repair/regeneration of the lung, or means to alter ECM composition to a more permissive state need to be developed.

## Supporting Information

S1 Fig Gene expression in native lungs from young and old BALB/c mice as assessed by qRT-PCR. N = 6/group; *p<0.05 old vs young. Values are normalized to a representative 3 wo mouse.
(TIF)

S2 Fig Gene expression (qRT-PCR) of hLFs after 1 week incubation in young and old decellularized BALB/c mouse lung matrix. Values were normalized to cells grown on tissue culture plastic. N = 6/group; *p<0.05 for 2 yo vs 4 wo.
(TIF)

S1 Supplemental Methods (DOCX)

S1 Table Antibodies used for immunofluorescence. (DOCX)

S2 Table Probes used for qRT-PCR. All probes were purchased from Life Technologies (Grand Island, NY).
(DOCX)

S3 Table Differential expression of proteins in young vs old decellularized mouse lungs using standard LC-MS/mass spectrometry. Average raw unique peptide hits for proteins positively identified in young (3 week) vs old (1 year) decellularized mouse lungs (n = 3/group).
(XLS)
