# Supplementary figures and images for: Decreased Laminin Expression by Human Lung Epithelial Cells and Fibroblasts Cultured in Acellular Lung Scaffolds from Aged Mice
Source: PLoS One. 2016 Mar 8;11(3):e0150966. doi: 10.1371/journal.pone.0150966 (PMC4783067; doi:10.1371/journal.pone.0150966)

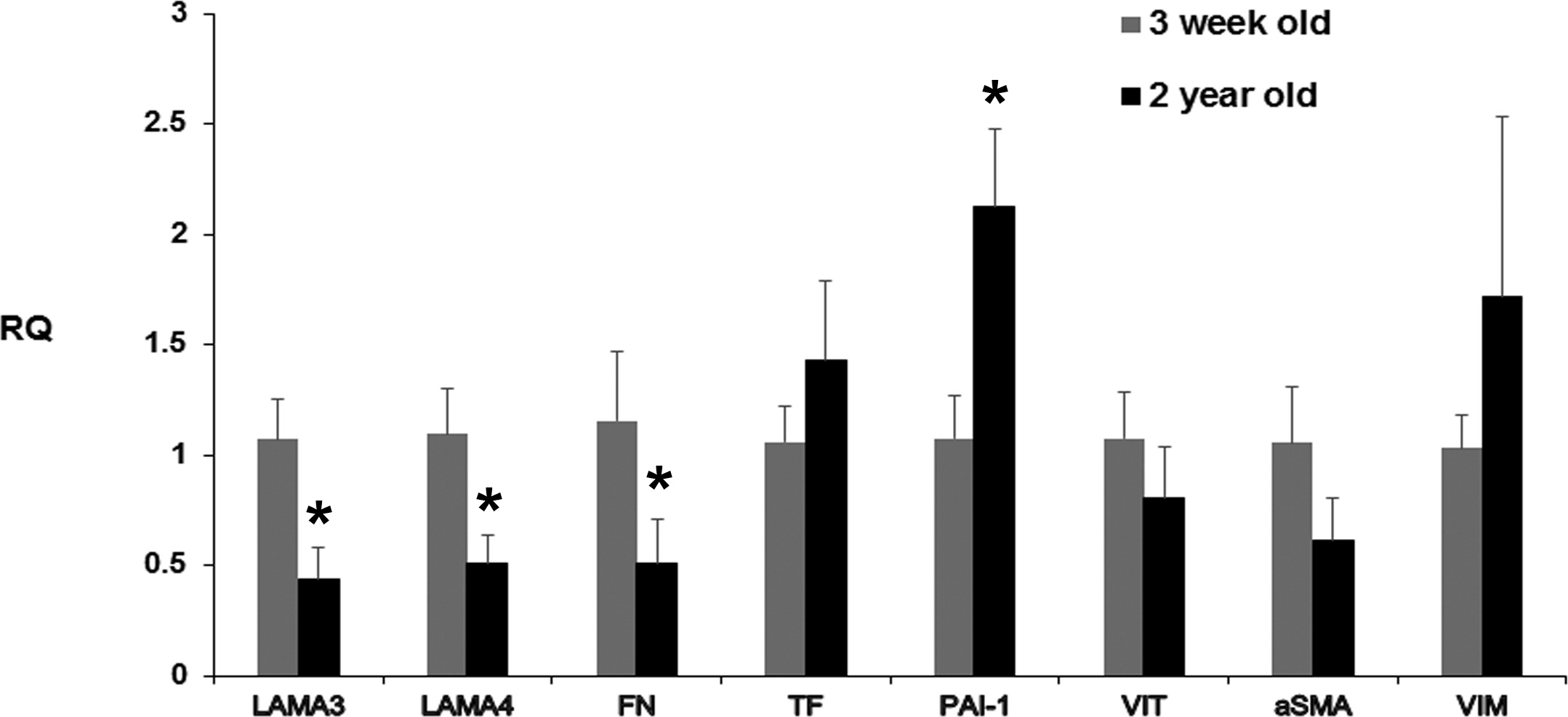

Supplement: S1 Fig — N = 6/group; *p<0.05 old vs young. Values are normalized to a representative 3 wo mouse. (TIF) [file pone.0150966.s001.tif]

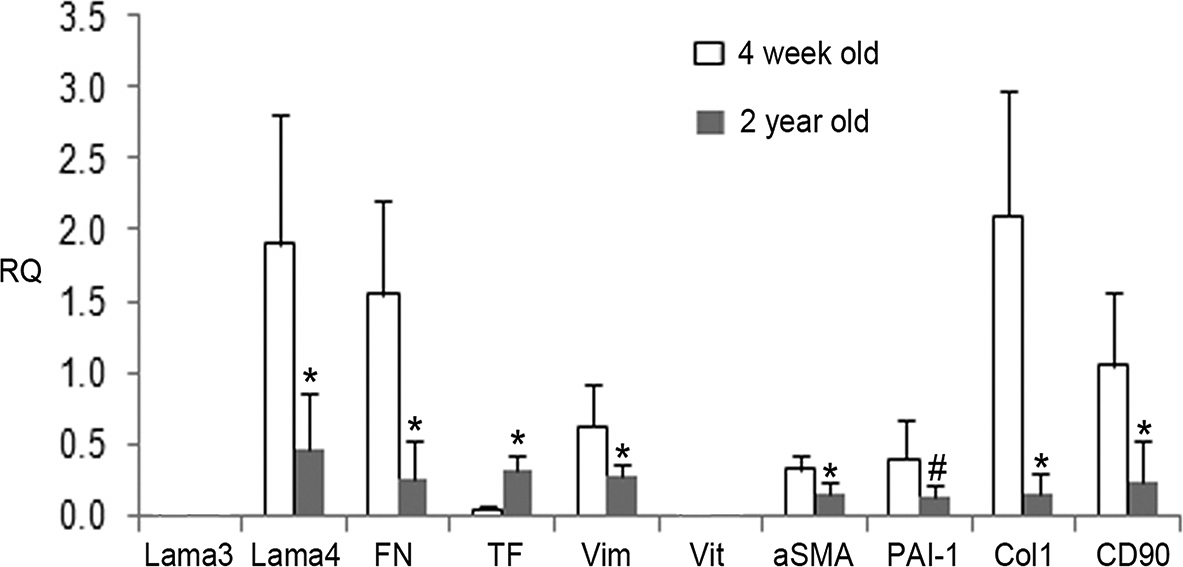

Supplement: S2 Fig — Values were normalized to cells grown on tissue culture plastic. N = 6/group; *p<0.05 for 2 yo vs 4 wo. (TIF) [file pone.0150966.s002.tif]
