# Supplementary material for: Decreased Laminin Expression by Human Lung Epithelial Cells and Fibroblasts Cultured in Acellular Lung Scaffolds from Aged Mice
Source: PLoS One. 2016 Mar 8;11(3):e0150966. doi: 10.1371/journal.pone.0150966 (PMC4783067; doi:10.1371/journal.pone.0150966)
Supplement: S1 Supplemental Methods — (DOCX) [file pone.0150966.s003.docx]

**Supplemental Methods**

Owing to methodological differences in protein identification using mass spectrometry proteomics[[1](#_ENREF_1)], both a standard 1D in-gel trypsin digestion method[[2](#_ENREF_2)], and iTRAQ[[3](#_ENREF_3)] method were used for proteomic analysis of decellularized young and old lung ECM.

**Unlabeled Mass Spectrometry (1D in-gel method)**

Decellularized inferior right lung lobes (approximately 125 mg for each sample) were homogenized using a Polytron and run on a NuPAGE Novex 4-12% Bis-Tris Page Gel and stained with coomassie blue. Seven individual bands were then excised from each sample and destained using 50mM ammonium bicarbonate (NH_4_HCO_3_) in 50% acetonitrile until excised bands were clear. Bands were then incubated for 30 minutes in 100% acetonitrile and then dried using a Speed Vac. Samples were then rehydrated with 100mM NH_4_HCO_3_ for 10 minutes and again dehydrated with 100% acetonitrile and dried with a Speed Vac to evaporate all remaining solvents. Gel samples were rehydrated in trypsin solution (1:100) and digested overnight in digestion buffer (40mM NH_4_HCO_3_ in 5% acetonitrile) at 37°C. Supernatants were then incubated for one hour in 5% formic acid, followed by a second incubation of the supernatants in 5% formic acid in 50% acetonitrile. Samples were then processed similarly as previously described[[2](#_ENREF_2)]. After centrifugation at 14,000 x g, 15μL of supernatant was desalted using a C18 ZipTip (P10; Millipore Corporation), dried again, and reconstituted in 20μL 0.1% formic acid and 2% acetonitrile. Samples were then loaded onto a 100 um x 120 mm fused silica microcapillary column packed with MAGIC C18 (5µm particle size, 20 nm pore size, Michrom Bioresources) at a flow rate of 500nL/min. Peptides were separated by a gradient comprising 3 – 60% ACN/0.1% formic acid and introduced into a linear ion trap (LTQ)-Orbitrap mass spectrometer (Thermo Fisher Scientific) via a nanospray ionization source. Mass spectrometry data was acquired in a data-dependent acquisition mode, in which an Orbitrap survey scan from m/z 400-2000 (resolution: 30,000 FWHM at m/z 400) was paralleled by 10 LTQ MS/MS scans of the most abundant ions.

The product ion spectra were searched against the IPI mouse database (v. 3.75) using SEQUEST (Bioworks 3.3.1, Thermo Fisher Scientific) exactly as described previously[[2](#_ENREF_2)]. A 1% false discovery rate was used. Protein identifications were ranked by number of peptides identified.

All IPI accession numbers were manually searched in the UniProtKB/Swiss Prot database (http://www.uniprot.org/help/about). Proteins were grouped according to their subcellular location – nucleus, cytoplasm, cytoskeletal, membrane, or extracellular matrix. If a protein was matched to more than one category, its predominant subcellular location was used for functional grouping. Protein identification was assigned based on two or more unique peptide hits within individual samples, not across samples.

**iTRAQ Mass Spectrometry**

Tissue Processing: Lung tissue samples (100 mg) washed in a PBS solution were placed in Eppendorf SafeLock™ tubes containing 75μL 2.0mm Zirconium Oxide beads, 50μL 1.0mm Zirconium Oxide beads, 50μL 0.5mm Zirconium Oxide beads with 6X sample volume lysis buffer consisting of 0.5M TEAB, 7M urea, 2M thiourea, 20% methanol and 4mM TCEP. The Bullet Blender Storm™ bead mill homogenizer was then utilized to homogenize the sample at 4^o^ C at maximum agitation for 10 minutes (Next Advance Averill Park, NY). Thorough lysis and membrane disruption was achieved through use of a Barocycler® NEP2320 capable of applying a maximum hydrostatic pressure of 35,000 psi. The samples underwent thirty cycles at 36^o^C, 35,000 psi for 30 seconds and ~0 psi for 10 seconds in PCT tubes with 150μL caps (Pressure Biosciences Inc. South Easton, MA). Methyl methanethiosulfonate (MMTS) was added to a final concentration of 8mM. The samples were transferred to a new 1.6 mL microfuge Eppendorf Protein LoBind tube and incubated 15 minutes at room temperature. The samples were then microfuged at 4^o^C, 12,000 x g for 10 minutes and the supernatant transferred to a new Eppendorf Protein LoBind microfuge tube.

Enzymatic (Tryptic) Digestion of Protein: Total protein concentrations were determined using Bradford assay (Bio-Rad, Hercules, CA). A 60μg aliquot of each sample was added to a new 1.5mL tube and all samples were brought to equal volume with sample lysis buffer without TCEP. Samples were then diluted four fold with mass spec grade water (Fisher W5SK-1 Pittsburgh, PA). Trypsin Gold (Promega Madison, WI) was added at a trypsin:total protein ratio of 1:30. Samples were incubated for 16 hours at 37^o^C, then frozen at -80^o^ C for 0.5 hours and dried in a speed vacuum centrifuge (Thermo Scientific Waltham, MA).

Peptide Purification: Dried peptides were resuspended in 1mL of washing solvent [98% water, 2% acetonitrile, 0.1% Trifluoroacetic acid (TFA)]. Resprep® (Restek, Bellafonte, PA) 3cc C18 cartridges were conditioned with 1mL 80% acetonitrile, 0.1% TFA, followed by 3mL of washing solvent. Samples were then added to the cartridge with a flow rate of approximately one drop per second (~1.7psi) followed by 3mL of washing solvent. Peptides were eluted in 1mL of 70% acetonitrile, 0.1% TFA and vacuum dried. Samples were then resuspended in 0.5M TEAB at a concentration of 2 μg/μL and a 1.5 μL aliquot was analyzed on a Linear Trap Quadrupole (LTQ) mass spectrometer (Thermo Scientific Waltham, MA) to check for proper peptide digestion and sample integrity.

Labeling and Detection: A 20μg aliquot of each processed sample was labeled with iTRAQ 8-plex reagents according to manufacturer’s protocol (ABSciex Framingham, MA). Intra-run comparison was achieved through normalization of samples to a pooled mastermix sample containing equal amounts of protein from the samples and labeled with the 119 and 121 tags. Following the labeling reaction, all samples and controls were multiplexed, vacuum dried and purified using a Resprep® 3cc MCX cartridge.

Offline Fractionation: The sample was resuspended in Buffer A (10mM ammonium formate, pH 10 in 98:2 water:acetonitrile) and fractionated offline by high pH C18 reversed-phase (RP) chromatography followed by fraction concatenation for 2D proteomic analysis. A MAGIC 2002 HPLC (Michrom BioResources, Inc., Auburn, CA) was used with a C18 Gemini-NX column, 150 mm x 2 mm internal diameter, 5µm particle, 110 Å pore size (Phenomenex, Torrence, CA). The flow rate was 150µl/minute with a gradient from 0-35% Buffer B (10 mM ammonium formate, pH 10 in 10:90 water:acetonitrile) over 60 minutes, followed by 35-60% over 5 minutes. Fractions were collected every 2 minutes and UV absorbance were monitored at 215 and 280 nm. Peptide containing fractions were divided into two equal numbered groups, “early” and “late”. The first “early” fraction was concatenated with the first “late” fraction, and so on. Concatenated samples were vacuum dried and resuspended in load solvent (98:2:0.01, water:acetonitrile:formic acid).

Online Fractionation and Data Acquisition: Digested peptide mixtures were desalted with C18 resin according to the stop and go procedure[[4](#_ENREF_4)]. Aliquots of 1-1.5µg of total peptide were dissolved in 5.5µL of load solvent A (98:2:0.01, water:acetonitrile:formic acid) and loaded directly onto a 12 cm X 75-µm internal diameter fused silica pulled-tip (New Objective Woburn, MA) capillary column packed in-house with MagicC18AQ resin (5 µm, 200 Å pore size; Michrom BioResources Auburn, CA) with load solvent at a flow rate of 800nL/min using an Eksigent 1D+LC nanoflow system (Dublin, CA) and a MicroAS autosampler. Peptides were eluted using a gradient of 10–40% B Solvent (98:2:0.01, acetonitrile:water:formic acid) over 55 at 320nL/min. The column was mounted in a nanospray source directly in line with a Velos Orbitrap mass spectrometer (Thermo Scientific Inc. Waltham, MA).

iTRAQ Statistical Analysis: Intra-run comparison was achieved through normalization of test samples to a pooled mastermix sample containing equal amounts of protein from each test sample. All processed samples were labeled with iTRAQ 8-plex reagent according to manufacturer’s protocol (ABSciex Framingham, MA).

Raw files obtained directly from the Orbitrap Velos XL Mass Spectrometer were imported into GalaxyP (See <https://usegalaxyp.org/> for public instance) for further processing (as described in z.umn.edu/ppingp). Within GalaxyP, all raw files were converted to .mzml format using msconvert and then into .mgf files using MGF formatter. Protein Pilot 4.5 search was performed with a target-decoy version database generated from the mouse UniProt database (8/1/2014) and ABSciex contaminant database.

False discovery rate (FDR) analysis employed the Proteomics System Performance Evaluation Pipeline Software within the ProteinPilot suite of software[[5](#_ENREF_5)]. All of the processing mentioned above was within the GalaxyP framework which offers highly-reproducible, robust, and easily-shareable workflows[[6](#_ENREF_6)]. The quantitative 1% FDR for each iTRAQ sample was calculated. Quantitative protein expression values with corresponding p-values conforming to 1% FDR or lower were analyzed using Ingenuity Pathways Analyses (IPA) Ingenuity® Systems, Qiagen, Valencia, CA.

References for Supplemental Information

1. Yates, J.R., C.I. Ruse, and A. Nakorchevsky, *Proteomics by mass spectrometry: approaches, advances, and applications.* Annu Rev Biomed Eng, 2009. **11**: p. 49-79.

2. Sokocevic, D., et al., *The effect of age and emphysematous and fibrotic injury on the re-cellularization of de-cellularized lungs.* Biomaterials, 2013. **34**(13): p. 3256-69.

3. Ross, P.L., et al., *Multiplexed protein quantitation in Saccharomyces cerevisiae using amine-reactive isobaric tagging reagents.* Mol Cell Proteomics, 2004. **3**(12): p. 1154-69.

4. Rappsilber, J., Y. Ishihama, and M. Mann, *Stop and go extraction tips for matrix-assisted laser desorption/ionization, nanoelectrospray, and LC/MS sample pretreatment in proteomics.* Anal Chem, 2003. **75**(3): p. 663-70.

5. Tang, W.H., I.V. Shilov, and S.L. Seymour, *Nonlinear fitting method for determining local false discovery rates from decoy database searches.* J Proteome Res, 2008. **7**(9): p. 3661-7.

6. Jagtap, P.D., et al., *Flexible and accessible workflows for improved proteogenomic analysis using the galaxy framework.* J Proteome Res, 2014. **13**(12): p. 5898-908.
