# Supplementary material for: Decreased Laminin Expression by Human Lung Epithelial Cells and Fibroblasts Cultured in Acellular Lung Scaffolds from Aged Mice
Source: PLoS One. 2016 Mar 8;11(3):e0150966. doi: 10.1371/journal.pone.0150966 (PMC4783067; doi:10.1371/journal.pone.0150966)
Supplement: S1 Table — (DOCX) [file pone.0150966.s004.docx]

| Analyte | Primary Antibody | Source | Secondary Antibody | Source |
| --- | --- | --- | --- | --- |
| Fibronectin (mouse) | Rabbit-anti-mouse fibronectin | Abcam, Cambridge MA | Alexa488-donkey-anti-rabbit | Jackson Immunoresearch, West Grove, PA |
| Vitronectin (mouse) | Rabbit-anti-mouse vitronectin | Cosmo Bio Co, Tokyo, Japan | Alexa488-donkey-anti-rabbit | Jackson Immunoresearch |
| Laminin-α3 (mouse&human) | Rabbit-anti-laminin α3 | Santa Cruz Biotechnology, Dallas, TX | Alexa488-donkey-anti-rabbit | Jackson Immunoresearch |
| Laminin-α3 (human only) | mouse-anti-human laminin α3 | R&D Systems, Minneapolis, MN | FITC-Goat-anti-mouse | Jackson Immunoresearch |
| Laminin-α4 (mouse) | Rat-anti-mouse laminin α4 | R&D Systems | Cy3-donkey-anti-rat | Jackson Immunoresearch |
| Laminin-α4 (human) | Sheep-anti-human laminin-α4 | R&D Systems | Alexa488-donkey-anti-sheep | Jackson Immunoresearch |
