# Supplementary material for: Decreased Laminin Expression by Human Lung Epithelial Cells and Fibroblasts Cultured in Acellular Lung Scaffolds from Aged Mice
Source: PLoS One. 2016 Mar 8;11(3):e0150966. doi: 10.1371/journal.pone.0150966 (PMC4783067; doi:10.1371/journal.pone.0150966)
Supplement: S2 Table — All probes were purchased from Life Technologies (Grand Island, NY). (DOCX) [file pone.0150966.s005.docx]

| Gene | Probe number (mouse) | Probe number (human) |
| --- | --- | --- |
| GAPDH | Mm99999915_g1 | Hs02758991_g1 |
| E-cadherin CDH1 |  | Hs01023894_m1 |
| N-cadherin CDH2 |  | Hs00983056_m1 |
| αCatenin CTNNA1 |  | Hs00944794_m1 |
| βCatenin CTNNB1 |  | Hs00355049_m1 |
| CD90 |  | Hs00174816_m1 |
| Collagen 1 alpha 1 |  | Hs00164004_m1 |
| Collagen 3 alpha 1 | Mm01254476_m1 | Hs00943809_m1 |
| Collagen 4 alpha 3 |  | Hs01022542_m1 |
| Elastin | Mm00514670_m1 | Hs00355783_m1 |
| Fibronectin | Mm01256744_m1 | Hs00365052_m1 |
| Lama3 | Mm01254735_m1 | Hs00165042_m1 |
| Lama4 | Mm01193660_m1 | Hs00935293_m1 |
| PAI-1 | Mm00435860_m1 | Hs01126607_g1 |
| α-Smooth Muscle Actin | Mm00725412_s1 | Hs00426835_g1 |
| Tissue Factor | Mm00438853_m1 | Hs01076029_m1 |
| Vimentin | Mm01333430_m1 | Hs00185584_m1 |
| Vitronectin | Mm00495976_m1 | Hs00940758_g1 |
